# Benign Fibrous Histiocytoma of the Conjunctiva

**DOI:** 10.1155/2012/786260

**Published:** 2012-12-23

**Authors:** Geoffrey Ryan, Bill Glasson, Andrew Foster

**Affiliations:** ^1^Ophthalmology Department, Caloundra Hospital, Sunshine Coast, West Terrace, Caloundra, QLD 4551, Australia; ^2^Terrace Eye Centre, Brisbane, QLD 4000, Australia; ^3^Golden Eye Clinic, Sunshine Coast, QLD 4551, Australia

## Abstract

A case is reported on recurrence of a rare conjunctival fibrous histiocytoma (FH) in a 38-year-old man. The lesion was excised and subsequently sent for histopathology and immunostaining resulting in a diagnosis of fibrous histiocytoma (FH). Recurrence was detected 3 months postoperatively requiring a conjunctivosclerokeratomy with cryotherapy and flap. At 6-month followup there was no recurrence detected.

## 1. Introduction

Fibrous histiocytomas are a heterogenous group of mesenchymal tumours composed of fibroblastic and histiocytic elements in varying proportions [1]. They often present as a solitary, slow growing, red nodule that most commonly affects the lower extremities, especially the thigh, followed by the upper extremities and retroperitoneum [2]. They can also involve ocular structures including the orbit, eyelids, conjunctiva, corneoscleral limbus, and cornea [1]. Whilst commonly found in the orbit, fibrous histiocytomas of the conjunctiva are exceedingly rare tumors [3]. This report describes the histological diagnosis and management of a recurrent conjunctival benign fibrous histiocytoma.

## 2. Case Presentation

**Figure 1:**
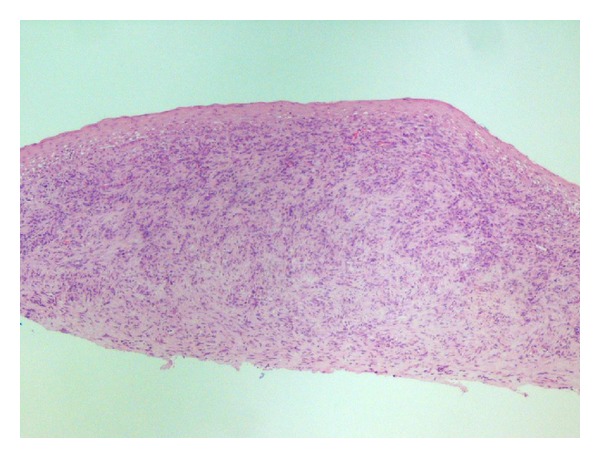
Low power showing a poorly demarcated tumour.

**Figure 2:**
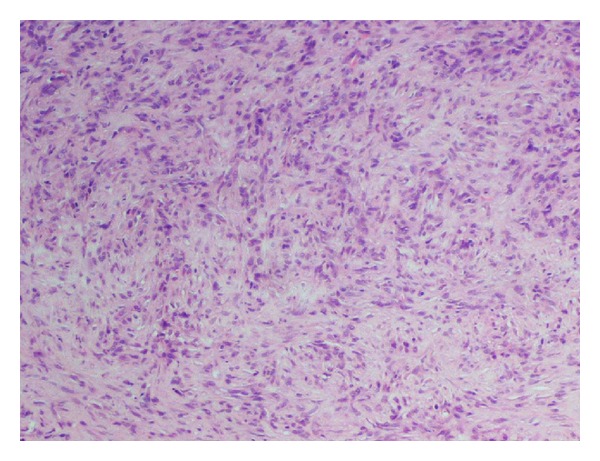
Microscopic high power showing spindle cells with focal storiform arrangement and lymphocytic infiltrate.

A 38-year-old male presented with an erythematous ocular surface lesion on the superior nasal aspect of the conjunctiva of the right eye, which had been present for three weeks. The patient had no prior ophthalmic or medical history. On examination the visual acuity using a Snellen chart was 6/6 bilaterally. The lesion was initially suspected to be a phlyctenule and the patient was trialled on dexamethasone eye drops (0.1%) four times a day for one week. After failing to resolve, the patient underwent surgical excision of the lesion. A piece of tissue excised measuring 5 × 3 × 1 mm was sent for histology. The specimen was confirmed to be conjunctiva with vascularised spindle cell proliferation within the stromal layer. At the margin of this proliferation was moderately intense lymphocytic infiltrate. Immunostaining showed the lesional cells to be negative for Keratin, S100, Melan A, and HMB45, excluding both epithelial and melanocytic differentiation. Further stains demonstrated the cells to be negative for CD4 but strongly positive for Factor XIIIa throughout much of the tumour. Based on the results of immunostaining, the diagnosis of a fibrous histiocytoma was confirmed (Figures 1 and 2).

Three months after surgical excision the lesion recurred. The patient subsequently underwent a right conjunctivosclerokeratomy with cryotherapy and flap. The second excision resulted in a crescent-shaped portion of pale tissue measuring 11 × 5 × 1 mm, which was shown to be conjunctiva. At the periphery of the limbal side there was a mild increase in stromal atypia and focal mild storiform arrangement. Staining was negative for CD4 and positive for factor XIIIa. The specimen contained residual fibrous histiocytoma along with features consistent with a hyperplastic scar. Six months following the second procedure there was no clinical evidence of recurrence.

## 3. Discussion

Conjunctival fibrous histiocytomas can resemble a variety of epibulbar conditions and in the past have been misdiagnosed as nodular episcleritis, juvenile xanthogranuloma, amelanotic melanoma, pterygium, carcinoma, inflammation, and leiomyoma [2]. Fibrous histiocytomas can be classified as benign or malignant [2]. The pivotal point in differentiating between benign fibrous histiocytoma (BFH) and pleomorphic undifferentiated sarcoma (previously known as malignant fibrous histiocytoma) is the histological presence or absence of pleomorphism and atypical mitotic activity [4]. Pleomorphic undifferentiated sarcomas have a local recurrence rate between 19% and 31%, with a metastatic rate at 31% to 35%, and a five-year survival reported at 65% to 70%. Both local recurrence and distant metastases can develop within one to two years from diagnosis, and the lung is the most common site of metastatic spread [2]. A literature review by Kim et al. reports, the mean age of diagnosis of a conjunctival FH was 39 years, with no gender predilection. The mean duration of symptoms before diagnosis was eight months [2]. The characteristic clinical features of fibrous histiocytoma are a history of recent onset, tan-yellow colour of the lesion, and absence of true feeder vessels unlike amelanotic melanomas, which possess true feeder vessels. Fibrous histiocytomas usually have vessels coursing over the surface of the tumour rather than entering at its base [5]. It has been suggested by Kim et al. that mitotic activity found in the tumor near the limbus may explain the propensity of FH to occur at the limbus, from which the corneal epithelial stem cells are believed to arise. 

The most appropriate management of FH at any site, particularly the conjunctiva, is complete surgical excision with tumor-free margins [2]. The lesion should be managed by resecting it with a 4 to 5 mm wide margin, planar base clearance, excision edge cryotherapy, excision base cryotherapy for planar invasion, and appropriate ocular surface reconstruction [5]. A pathologist should be consulted before and after surgery to ensure that appropriate measures are taken to care for the specimen. Furthermore, the surgeon must ensure the specimen is clearly orientated to allow the pathologist to confidently report on the excision edge and base clearance. In the event of positive margins, adjuvant treatment such as brachytherapy is recommended [2]. In order to exclude disease recurrence, patients require close, lifelong followup. 

## References

[1] Lahoud S, Brownstein S, Laflamme MY (1988). Fibrous histiocytoma of the corneoscleral limbus and conjunctiva. *American Journal of Ophthalmology*.

[2] Kim HJ, Shields CL, Eagle RC, Shields JA (2006). Fibrous histiocytoma of the conjunctiva. *American Journal of Ophthalmology*.

[3] Gupta VP, Saxena T, Dev G (2002). Fibrous histiocytoma in primary pterygium. *Orbit*.

[4] Akbulut S, Arikanoglu Z, Basbug M (2012). Benign fibrous histiocytoma arising from the right shoulder: is immunohistochemical staining always required for a definitive diagnosis?. *International Journal of Surgery Case Reports*.

[5] Arora R, Monga S, Mehta DK, Raina UK, Gogi A, Gupta SD (2006). Malignant fibrous histiocytoma of the conjunctiva. *Clinical and Experimental Ophthalmology*.

